# Current Research Therapeutic Strategies for Alzheimer's Disease Treatment

**DOI:** 10.1155/2016/8501693

**Published:** 2016-01-03

**Authors:** Jaume Folch, Dmitry Petrov, Miren Ettcheto, Sonia Abad, Elena Sánchez-López, M. Luisa García, Jordi Olloquequi, Carlos Beas-Zarate, Carme Auladell, Antoni Camins

**Affiliations:** ^1^Unitat de Bioquímica, Facultat de Medicina i Ciències de la Salut, Universitat Rovira i Virgili, C./St. Llorenç 21, Tarragona, 43201 Reus, Spain; ^2^Biomedical Research Networking Center in Neurodegenerative Diseases (CIBERNED), Madrid, Spain; ^3^Unitat de Farmacologia i Farmacognòsia Facultat de Farmàcia, Institut de Biomedicina (IBUB), Universitat de Barcelona, 08028 Barcelona, Spain; ^4^Department of Physical Chemistry, Faculty of Pharmacy, University of Barcelona, 08028 Barcelona, Spain; ^5^Facultad de Ciencias de la Salud, Universidad Autónoma de Chile, 3460000 Talca, Chile; ^6^Departamento de Biología Celular y Molecular, C.U.C.B.A., Universidad de Guadalajara and División de Neurociencias, Sierra Mojada 800, Col. Independencia, 44340 Guadalajara, JAL, Mexico; ^7^Centro de Investigación Biomédica de Occidente (CIBO), Instituto Mexicano del Seguro Social (IMSS), 44340 Guadalajara, JAL, Mexico; ^8^Departament de Biologia Cel.lular, Facultat de Biologia, Universitat de Barcelona, 08028 Barcelona, Spain

## Abstract

Alzheimer's disease (AD) currently presents one of the biggest healthcare issues in the developed countries. There is no effective treatment capable of slowing down disease progression. In recent years the main focus of research on novel pharmacotherapies was based on the amyloidogenic hypothesis of AD, which posits that the beta amyloid (A*β*) peptide is chiefly responsible for cognitive impairment and neuronal death. The goal of such treatments is (a) to reduce A*β* production through the inhibition of *β* and *γ* secretase enzymes and (b) to promote dissolution of existing cerebral A*β* plaques. However, this approach has proven to be only modestly effective. Recent studies suggest an alternative strategy centred on the inhibition of the downstream A*β* signalling, particularly at the synapse. A*β* oligomers may cause aberrant N-methyl-D-aspartate receptor (NMDAR) activation postsynaptically by forming complexes with the cell-surface prion protein (PrPC). PrPC is enriched at the neuronal postsynaptic density, where it interacts with Fyn tyrosine kinase. Fyn activation occurs when A*β* is bound to PrPC-Fyn complex. Fyn causes tyrosine phosphorylation of the NR2B subunit of metabotropic glutamate receptor 5 (mGluR5). Fyn kinase blockers masitinib and saracatinib have proven to be efficacious in treating AD symptoms in experimental mouse models of the disease.

## 1. Introduction

Alzheimer's disease (AD) is currently incurable neurodegenerative condition which is highly prevalent in old age [1–3]. It was first described in 1906 by Alois Alzheimer, who analysed brain tissue from a patient who had died from an unknown mental illness. According to the Alzheimer's Association, 13% of people over 65 suffer from this disease in developed countries, where it is the fifth leading cause of death in patients at this age. According to the World Health Organization (WHO) estimates, the overall projected prevalence in global population will quadruple in the next decades, reaching 114 million patients by 2050 [2]. Apart from having a great social impact, this would clearly lead to increased economic burden to healthcare systems worldwide [1–3].

AD is classified according to the age of onset and whether it is developed spontaneously or as a result of genetic mutations. Familial AD (FAD) is an early-onset (sometimes as early as 40 years of age) disease, which is caused by hereditary mutations and represents approximately 2% of diagnosed cases. The vast majority of patients suffer from the sporadic AD, which is subdivided into early- and late-onset forms. If identified in individuals under 65 years of age, early-onset diagnosis is given (3–5% prevalence), with the rest of the cases referred to as a late-onset AD (95–97% prevalence) [3–7]. In FAD, mutations in genes coding for amyloid precursor protein (APP; chromosome 21), presenilin 1 (PS1; chromosome 14) and presenilin 2 (PS2; chromosome 1), serve as triggers for beta amyloid (A*β*) formation, particularly of the long form of the peptide (A*β*1-42). In case of sporadic AD, a significant number of patients (approximately 25%) are carriers of the e4 allele of the ApoE gene (apolipoprotein E; chromosome 19), a lipid transport protein. The exact mechanism whereby ApoE contributes to increased A*β* levels is currently unknown [6–9].

Aging is considered the principal risk factor for sporadic AD development. Other potential risk factors including hypertension, dyslipidemia, metabolic syndrome and diabetes have also been identified [10–12].

In the present paper, we discuss treatment strategies structured according to a number of existing hypotheses aimed at explaining the origins of AD:

- amyloid cascade hypothesis,
- cholinergic hypothesis,
- dendritic hypothesis,
- mitochondrial cascade hypothesis,
- metabolic hypothesis,
- other hypotheses (oxidative stress, neuroinflammation).

The principal targets and clinical trials of the compounds aimed at reducing A*β* formation and plaques are summarized in Table 1. Relevant data for the molecules developed in the context of cholinergic, dendritic, mitochondrial cascade, metabolic and other hypotheses are presented in Table 2.

## 2. The Amyloid Cascade Hypothesis

A*β* peptide is derived from proteolysis of APP, an integral transmembrane protein found in different cell types, including neurons and glial cells [1–4]. In humans, alternative splicing produces multiple isoforms of the molecule, with APP695 being the most abundant in the brain [3]. APP is processed into smaller peptide fragments, one of which is A*β*, via cleavage by *α*-, *β*-, and *γ*-secretase enzyme protein complexes, which include presenilin and nicastrin molecules [8]. Under physiological conditions, APP is catabolized by the *α*-secretase to produce a soluble sAPP*α* fragment, which remains in the extracellular space, and a carboxy-terminal 83-amino acid (C83) fragment, which is anchored in the plasma membrane [8–10]. sAPP*α* is involved in the regulation of neuronal excitability, improves synaptic plasticity, learning, and memory, and increases neuronal resistance to oxidative and metabolic stresses [8]. In a neuropathological situation, APP is first preferentially cleaved by *β*-secretase 1 (BACE), which fragments APP into sAPP*β* and a 99-amino acid membrane-bound fraction (C99). Additional processing of the C99 fragment by *γ*-secretase results in the generation of either A*β*(1-40) or A*β*(1-42) peptides, thought to be responsible for senile plaque formation [8–12]. Whilst sAPP*α* is beneficial to the organism, A*β* peptides may cause synaptic loss, decrease neuronal plasticity, alter energy metabolism, induce oxidative stress and mitochondrial dysfunction, and may provoke disruptions in cellular calcium homeostasis [8, 9].

The amyloid cascade hypothesis suggests that the formation, aggregation, and deposition of A*β* peptides, and especially A*β*(1-42), are a primary event in AD pathogenesis which triggers neurotoxicity and neurodegeneration [6–8]. Excessive extracellular A*β* may also presumably lead to increased Tau phosphorylation and the formation of neurofibrillary tangles. Molecular genetics studies into the mechanisms of FAD gave credence to this hypothesis, suggesting potential novel therapeutics, such as inhibitors of *β*- and *γ*-secretase or enhancers of *α*-secretase activity. However, in cases of sporadic AD, where A*β* generation does not appear to have a clear genetic basis, amyloid cascade hypothesis cannot fully explain the root causes of the disease [11–13].

### 2.1. Imbalance in the Generation/Removal of *β*-Amyloid in Alzheimer's Disease

*Role of Neuroinflammation*. It is believed that A*β* is generated continuously and its aggregation and subsequent plaque deposition in AD is concentration-dependent. Excessive accumulation of both soluble and insoluble A*β* may occur not only as a result of aberrant APP processing by *β*- and *γ*-secretase enzymes but may also be caused by inefficient removal of newly generated A*β*. Reduced activity of A*β*-degrading enzymes, such as neprilysin, insulin degrading enzyme (IDE), and angiotensin converting enzyme I (ACE I), may provoke an imbalance between the amyloid generation and clearance [13–19]. Additional predisposing factors, including the ApoE status and the presence of comorbidities such as metabolic syndrome and diabetes likely contribute to sporadic AD in a manner, which is still poorly understood.

A lack of direct correlation between amyloid plaque burden and memory loss in AD patients demonstrates that neurotoxicity is not solely dependent on the insoluble A*β* [14–16]. In fact, biochemical studies have demonstrated a good correlation between the levels of soluble A*β* oligomers in the brains of patients with AD and the degree of cognitive impairment [19]. It has been suggested that soluble A*β*-driven synaptic loss may be responsible for neurodegeneration observed in AD. If that turns out to be the case, then central nervous system (CNS) inflammatory processes will likely be implicated [20, 21]. Neuroinflammation is a blanket term used to describe immune response in neurodegenerative diseases. It involves the activation of glial cells, especially microglia and astroglia. Under physiological conditions, microglial cells have a phagocytic function. In AD, activated microglia secrete a large number of molecules [21–23]. Such substances, among which are proinflammatory cytokines, prostaglandins, reactive oxygen species (ROS), and nitric oxide synthase (NOS), contribute to a chronic state of perpetual stress. A prolonged release of all these factors can eventually cause neuronal death [22, 23].

### 2.2. Antiamyloidogenic Pathway and Amyloidogenic Route as Strategies for Development of Therapeutic Treatments Modifying the Course of Alzheimer's Disease

In the last two decades, the pharmaceutical industry has focused primarily on the amyloidocentric approach, devoting substantial resources to develop effective AD drugs. However, multiple failures of drug candidates in clinical trials have led researchers to question the feasibility of this strategy [10–12]. One possible reason for failure is a lack of biomarkers which could reliably identify AD in relatively early stages. It is entirely possible that the patients currently recruited for phase III trials are in such advanced stages of AD that any attempted intervention is probably useless. Therefore, new diagnostic tools capable of early detection are sorely needed. In the meantime, there is still a number of novel treatments under development, which target the amyloidogenic route. In order to reduce A*β* generation from the APP, *γ*- and *β*-secretase inhibition and the potentiation of *α*-secretase activity have been considered.

*Inhibitors and Modulators of β-Secretase*. *β*-secretase enzyme complex participates in the initial stages of the amyloidogenic APP-processing pathway. The development of *β*-secretase inhibitors is a challenge because, besides the APP, this complex has many more substrates. To give just one example, neuregulin-1, which is involved in the myelination of CNS axons and synaptic plasticity, is a target of *β*-secretase [3]. Broad range of substrates can lead to significant side effects, even if the specific inhibition of the enzyme is achieved. Nevertheless, E2609 (clinical trial ID# NCT01600859), MK-8931 (NCT01739348), and LY2886721 (NCT01807026 and NCT01561430) have all shown efficacy in reducing A*β* production by up to 80–90% in the cerebrospinal fluid (CSF) in humans. None of *β*-secretase inhibitors have reached the market so far [3, 24–27].

*Inhibitors and Modulators of γ-Secretase*. The *γ*-secretase complex is responsible for the final stage of amyloidogenesis, leading to the generation of A*β*(1-40) and A*β*(1-42). *γ*-secretase inhibition was initially considered a promising disease-modifying strategy. However, substrate promiscuity presents similar issues facing *β*-secretase inhibitors [28–30]. Notch protein, responsible for regulating cell proliferation, development, differentiation, and cellular communication, is one of the targets of the *γ*-secretase [28]. Just as with the *β*-secretase inhibitors, off-target secondary effects are a major concern [30].

Semagacestat (LY450139) is a *γ*-secretase inhibitor that decreased A*β* levels in blood and CSF in humans [31]. The clinical study results, (NCT00762411, NCT01035138, and NCT00762411) which recruited over 3000 patients, are an example of the worst possible outcomes. It was reported that semagacestat treatment was associated with the worsening of cognition and the abilities to carry out the activities of daily living (ADAS-cog scale) in AD patients. Additional side effects included weight loss, increased incidence of skin cancer, and a higher risk of infection. Avagacestat is another *γ*-secretase inhibitor the development of which was discontinued as a result of a lack of efficacy (NCT00810147, NCT00890890, NCT00810147, NCT01079819) [32–34].

Selective *γ*-secretase modulators (SGSM) may, in theory, be developed in such a way as to avoid the adverse events associated with general enzyme inhibition. The goal of such treatments is to block APP processing without interfering with other signaling pathways like Notch [35].

SGSM development began with the observation that several nonsteroidal anti-inflammatory drugs (NSAIDs) decreased A*β*(1-42) peptide levels* in vitro* and* in vivo*. Examples of these drugs are ibuprofen, sulindac, indomethacin, and flurbiprofen [36]. The accepted mechanism of action (MOA) of NSAIDs is the inhibition of cyclooxygenase (COX) enzymes. While ibuprofen is a COX inhibitor, R-flurbiprofen (Tarenflurbil) is not, and its effects on the reduction of A*β* levels cannot be attributed to COX inhibition. Unfortunately, Tarenflurbil and Ibuprofen did not show efficacy for AD treatment in their respective clinical trials [36, 37]. CHF5074, just like R-flurbiprofen, is an NSAID devoid of COX inhibitory activity.* In vitro*, CHF5074 inhibited A*β*(1-42) production presumably by blocking *γ*-secretase complexes [38–41]. Recent studies have reclassified this compound as a microglial modulator based on its ability to reduce both amyloid burden and microglial activation [39]. Results from a phase II trial in patients with Mild Cognitive Impairment (MCI) indicate that CHF5074 treatment leads to improvements of several cognitive measures and reduces inflammatory marker levels in the CSF [38–40].

The idea that the long-term use of NSAIDs could confer some protection against AD generated some interest in NSAIDs as a treatment potentially useful for reducing A*β*(1-42) levels. However, negative results reported in clinical trials with NSAIDs suggest that this hypothesis requires further refinement [37].

Another example of a possible SGSM is NIC5-15, which is a naturally occurring molecule. NIC5-15, also known as pinitol, is a natural cyclic sugar alcohol [41]. This compound supposedly modulates *γ*-secretase and is reportedly capable of reducing A*β* production, while not affecting the substrate cleavage of Notch. No peer-reviewed data are available for this compound, so any reported results should be considered as a forward-looking statements requiring rigorous scientific proof. However, it is claimed that the compound improves cognitive function and memory in preclinical models of AD neuropathology. If true, these data suggest that NIC5-15 may be a suitable therapeutic agent for the treatment of AD for two reasons: (a) it preserves Notch activity and (b) also it is potentially an insulin sensitizer. Moreover, it is supposedly being investigated as an anti-inflammatory inhibitor because it may prevent microglia activation. Once again, independent researchers have not yet confirmed these results.

### 2.3. Inhibition of *β*-Amyloid Peptide Aggregation

A*β* peptide aggregates give rise to amyloid plaques. The following compounds were developed in order to prevent senile plaque formation.

The only inhibitor of A*β* aggregation that reached phase III trials is the 3-amino-1-propaneosulfonic acid (3-APS, Alzhemed, tramiprosate) [42, 43]. This medication was designed to interfere with or antagonize the interaction of soluble A*β* with endogenous glycosaminoglycans. Glycosaminoglycans have been shown to promote aggregation of A*β* amyloid fibril formation and deposition [43]. However, the disappointing results of the phase III clinical trial in 2007 have led to the suspension of this compound in Europe [44].

Colostrinin, a complex of proline-rich polypeptides present in ovine, bovine, and human colostrum inhibits aggregation of A*β* and its neurotoxicity in cell assays, and improves cognitive performance in mice models [45]. Although a phase II trial showed slight improvements in Mini Mental State Evaluation in patients with mild AD in a treatment period of 15 months, this beneficial effect was not maintained after another 15 months of continuous treatment [45].

Scyllo-inositol (ELND005) is an oral amyloid antiaggregation agent capable of reducing A*β* toxicity in the mouse hippocampus. 18-month long phase II clinical trial with ELND005 was conducted in participants with mild-to-moderate AD. This dose-finding, safety and efficacy trial did not meet its primary clinical efficiency outcomes [46].

Clinical trials for AD treatment were also performed with metal chelating 8-hydroxiquinolines (8-HQ) compounds clioquinol and PBT2 [47]. While their mechanism of action is not completely understood, it is thought that these molecules block the interaction between the base metals and brain A*β* peptide. It was suggested that increased levels of oxidative stress in the brain of AD patients might be partially due to copper ions binding to A*β*, leading to metal-mediated generation of reactive oxygen species (ROS) [48–50]. It was also hypothesized that 8-HQs may prevent A*β* aggregation while simultaneously restoring homeostasis in cellular levels of copper and zinc ions [49, 50]. Unfortunately, these molecules failed in the phases II and III of clinical development due to lack of efficacy.

### 2.4. Compounds Which Promote the Removal of Amyloid Deposits and Aggregates

Another potential treatment option which is centered on the amyloidogenic pathway is to promote the clearance of existing amyloid aggregates and deposits. To achieve this, three different strategies have been evaluated.

#### 2.4.1. Activation of Enzymes That Degrade Amyloid Plaques

Aggregates and amyloid plaques are degraded by multiple proteases including neprilysin, IDE, plasmin, endothelin converting enzyme, angiotensin converting enzyme, and metalloproteinases. Protein levels of these enzymes decrease in AD, which contributes to the formation and accumulation of A*β* [13–16]. Despite being an attractive strategy for developing disease-modifying drugs, no compounds with this MOA have ever reached advanced clinical development due to the lack of specificity.

#### 2.4.2. Modulation of *β*-Amyloid Transport between the Brain and the Peripheral Circulation

A*β* transport between the CNS and the peripheral circulation is regulated by (1) apolipoproteins (e.g., A*β* may be transported from the blood to the brain when it is bound to APOE); (2) low-density lipoprotein receptor-related protein (LRP-1), which increases A*β* outflow from the brain to the blood; (3) receptor for advanced glycation end products (RAGE), which facilitates the transport of A*β* across the blood-brain barrier (BBB) [15, 51, 52].

The goal of any treatment, which is focused on this mechanism, is to reduce cerebral amyloid load by attempting to restrict A*β* to the peripheral circulation. To this end, a number of different strategies have been proposed, notably the peripheral administration of LRP-1. However, the only drug candidates that have reached clinical stage are the inhibitors/modulators of RAGE. These include PF-0449470052, which failed in phase II trials, and TTP4000, with the phase I trial completed in February 2013 (NCT01548430). The results of this trial have not been published.

#### 2.4.3. Antiamyloid Immunotherapy

*Active Immunotherapy*. Immunotherapy strategy aimed to promote A*β* clearance with the objective of reducing the amyloid load in AD. Active immunization (vaccination) with either A*β*(1-42) (predominant form found in senile plaques) or other synthetic fragments has been successfully evaluated in transgenic mouse models of AD. Assays are generally based on the stimulation of B cells, T cells, and immune responses through activation of the phagocytic capacity of microglia. Human tests were initially promising; however treatment with the first-generation vaccine (AN1792) has produced serious adverse events that led to the discontinuation of the phase II trial. AN1792 consisted of a synthetic full-length A*β*(1-42) peptide with a QS-21 adjuvant. As a result of a T cell-mediated autoimmune response, 6% of patients have developed cerebral inflammation which turned out to be aseptic meningoencephalitis [53].

Second-generation vaccines were designed using a shorter A*β*(1-6) peptide segment in an attempt to prevent nonspecific immune response seen with the full-length vaccine. CAD 106, designed by Novartis, was the first second-generation vaccine that reached the clinical phases of development [54]. A recently completed phase II clinical trial have shown a A*β*-specific antibody response in 75% of treated patients, without causing adverse inflammatory reactions. ACC-001, developed by Janssen, has recently completed two-phase II trials (NCT01284387 and NCT00479557) with an additional phase II trial still ongoing (NCT01227564). However, the pharmaceutical company has abandoned the plans for further development of this vaccine. Other vaccines, including tetra-palmitoylated A*β*(1-15) reconstituted in a liposome (ACI-24), MER5101 and AF205 are currently in various stages of preclinical development [55–58].

*Passive Immunization*. It is the administration of monoclonal or polyclonal antibodies directed against A*β*. This therapy consists of the intravenous administration of anti-A*β* antibodies to the patient. An advantage of this strategy compared to active immunization is that the proinflammatory T cell-mediated immune reactions should not occur. Studies in transgenic animals have shown that passive immunization reduces cerebral amyloid load and improves cognition, even when the amyloid plaque numbers are not significantly reduced. This could be attributed to the neutralization of soluble amyloid oligomers, which are increasingly recognized to play a fundamental role in the pathophysiologic cascade of AD.

Bapineuzumab and solanezumab are two monoclonal antibodies that have reached advanced stages of clinical development [59]. However, in 2012, two phase III clinical trials had failed because of a lack of efficacy in patients with mild-to-moderate AD. Both bapineuzumab and solanezumab are humanized monoclonal antibodies against A*β*(1-6) and A*β*(12-28), respectively [60, 61]. In case of bapineuzumab, significant reduction in brain amyloid plaques and phosphorylated Tau in cerebrospinal fluid was reported. However, the treatment failed to produce significant improvements of cognitive function. In a solanezumab trial, infusions of 400 mg of solanezumab or placebo were administered once a month for 80 weeks in patients with mild-to-moderate AD. The results suggested that solanezumab may improve cognition in mild AD; however statistical significance was not achieved in that study [61]. Currently solanezumab is in phase III trials in patients with AD (NCT01127633 and NCT01900665) and in older individuals who have normal thinking and memory function but who may be at risk of developing AD in the future (NCT02008357).

Another monoclonal antibody, gantenerumab, is being investigated in people at risk of developing presenile AD due to genetic mutations. NCT01760005 trial is still recruiting participants and will determine the efficacy of both gantenerumab and solanezumab in the prodromal disease stages [62–64]. In parallel, two additional phase III trials of gantenerumab in patients with mild AD (NCT02051608) and prodromal AD (NCT01224106) are ongoing. Gantenerumab is a fully human IgG1 antibody designed to bind with high affinity to a conformational epitope on the *β*-amyloid fibres. Microglia recruitment and ensuing phagocytosis will presumably lead to amyloid plaque degradation. Experimental studies in transgenic mice support this hypothesis.

Crenezumab (MABT5102A) is a humanized monoclonal antibody which uses IgG4 backbone [65]. A phase II clinical trial to assess the safety and efficacy in patients with mild-to-moderate AD (NCT01343966) was completed in April 2014, although the results are not yet publicly available. The most recent phase II trial aiming to evaluate the safety and efficacy of crenezumab in asymptomatic carriers of E280A autosomal-dominant mutation of PSEN1 commenced in November 2013 (NCT01998841).

Other monoclonal antibodies against A*β* developed so far include PF-04360365 (ponezumab) which targets the free carboxy terminal amino acids 33–40 of the A*β* peptide; MABT5102A, which binds to A*β* monomers, oligomers, and fibrils with equally high affinity; GSK933776A, which is similar to bapineuzumab in that it binds to the N-terminal A*β*(1-5). In addition, other passive immunotherapies mostly in phase I clinical development include NI-101, SAR-228810, and BAN-2401 [57, 58, 61–65].

Gammagard is a preparation of antibodies from human plasma. Its safety for human use had been previously demonstrated in certain autoimmune conditions. Gammagard effects were evaluated in a small number of AD patients (NCT00818662). It is believed that this mixture contains a small fraction of polyclonal antibodies against the A*β* peptide. In addition, this preparation may possess immunomodulatory properties that could potentially enhance microglial phagocytosis [66–68].

## 3. Strategies Focused on Tau Proteins

Tau proteins are highly soluble and abundant in the neurons where they play a critical role in microtubule stabilization, particularly in axons [69–71]. Tau hyperphosphorylation leads to the formation of insoluble paired helical filaments (PHF) which form neurofibrillary tangles. The loss of microtubule-binding capacity provokes cytoskeleton destabilization, which eventually causes neurodegeneration and neuronal death [70]. As an alternative to amyloidocentric approaches, Tau-centered treatments aim to inhibit the phosphorylation and/or aggregation of Tau protein. In addition, microtubule-stabilizing drugs could be used as a disease-modifying strategy in AD [71]. In recent years, immunomodulation was suggested as a viable option for promoting effective clearance of Tau aggregates.

### 3.1. Inhibitors of Tau Hyperphosphorylation

All Tau proteins are a product of alternative splicing of a microtubule-associated protein Tau (MAPT) gene. Phosphorylation is the primary mechanism which regulates Tau binding to microtubules. Under physiological conditions the protein remains soluble; however, in AD, pathological hyperphosphorylation of Tau compromises its normal functions [72, 73]. Hyperphosphorylation occurs as a result of an imbalance between the catalytic activity of kinases and phosphatases. Increased expression of active forms of various kinases in the areas proximal to neurofibrillary tangles has been described in AD, including CDK5, GSK3*β*, Fyn, stress-activated protein kinases JNK and p38, and mitogen-activated protein kinases ERK1 and ERK2 [74]. Some or all of these kinases contribute to the perpetuation of the phosphorylation of Tau in neurofibrillary tangles [73, 75–81]. As a result, significant research efforts have been devoted to the development of kinase inhibitors as a possible treatment strategy for AD. For example, SP600125, a widely used pan-JNK inhibitor, exerts beneficial effects on cognition and reduces neurodegeneration in an APP/PS1 transgenic mouse model of AD [80]. It has been proposed that specific inhibition of JNK3 could be sufficient to bring similar benefits as seen with SP600125 in rodent models [78–81]. Human data in AD patients indicate a positive correlation between the levels of JNK3 and A*β*(1-42) in the brain [77]. Furthermore, JNK3 upregulation was detected in the CSF and was associated with memory loss. Thus, JNK3 inhibition remains a promising target for future therapies [81].

CDK5 belongs to the family of serine/threonine cyclin-dependent kinases and is responsible for a number of physiological functions within the CNS, including neurite outgrowth and the regulation of axonal development [82]. CDK5 catalytic activity is dependent on its direct association with p35, key regulator of CDK5 signaling. This cofactor is cleaved by a nonlysosomal protease calpain in a calcium-dependent manner [83]. Conversion of p35 to p25 results in prolonged activation and mislocalization of CDK5. Due to the increases in intracellular calcium levels observed in the brains of AD patients, pathological activation of CDK5 occurs, resulting in hyperphosphorylation of Tau and neuronal cell death [83, 84]. CDK5 inhibition may thus also be potentially considered as a possible drug target. Currently existing CDK5 inhibitors roscovitine and flavopiridol have demonstrated neuroprotective properties in* in vitro* and* in vivo* models of excitotoxicity, ischemia, and neurodegeneration [84, 85].

GSK3*β* inhibitors are arguably in the most advanced stages of clinical development for AD. Among the various drugs that are currently being studied, tideglusib, an irreversible inhibitor of GSK3*β*, has recently completed phase II trials (NCT01350362). Tideglusib administration for a period of 26 weeks to patients with mild-to-moderate AD did not show clinical efficacy, and the compound has since been discontinued for this indication [86]. Another study (NCT00948259) evaluated the safety and tolerability of a 20-week administration of NP031112 compared with placebo in patients with AD. No data has been reported for this study.

Phosphatase activation has also been considered as a possible drug target. Currently, there is only one protein phosphatase 2 (PP2A) agonist in development. Sodium selenite (VEL015) is undergoing phase II trials in Australia (ACTRN12611001200976). Experimental studies have shown that sodium selenate reduces Tau phosphorylation, both in cell culture and in mouse models of the disease [86–88]. VEL015 administration to rodents have resulted in significant cognitive improvements and substantial reduction of neurodegenerative phenotype.

### 3.2. Inhibitors of Tau Aggregation

Hyperphosphorylated Tau aggregates contribute to neurotoxicity observed in AD brain. Methylene blue dye derivatives have shown some promise in inhibiting the formation of Tau aggregates. Methylene blue disrupts the aggregation of Tau, has the ability to inhibit amyloid aggregation, improves the efficiency of mitochondrial electron transport chain, reduces oxidative stress, prevents mitochondrial damage, and is also a modulator of autophagy [74, 89]. The first-generation molecule derived from methylene blue (Rember) appeared to stabilize AD progression in a clinical trial which lasted 50 weeks. These results motivated researchers to develop a next-generation version of methylene blue, TRx 0237. This compound is a purified derivative of methylene blue which not only inhibits Tau protein aggregation but also dissolves brain aggregates of Tau [74]. Several clinical trials are currently underway (NCT01626391, NCT01689233, NCT01689246, NCT01626378) to evaluate the potential efficacy of this drug in AD.

### 3.3. Microtubule Stabilizers

Microtubule stabilization may potentially achieve a similar end-result as that seen with the inhibitors of Tau hyperphosphorylation and aggregation. Paclitaxel is a microtubule-stabilizing drug currently in use in the oncology field. Unfortunately, this compound is incapable of crossing the BBB and its use is associated with serious adverse events, which limits its utility in AD [90, 91]. In addition to paclitaxel, other microtubule-stabilizing compounds such as TPI 287 have been considered as a possible AD therapy. TPI 287 is a derivative of taxane, also used in cancer treatment. TPI 287 stabilizes the microtubules by binding to tubulin. NCT01966666 clinical trial will evaluate TPI-287 safety, pharmacokinetic properties, and tolerability by intravenous infusion in mild-to-moderate AD.

Epothilone D is a microtubule-stabilizing compound which improved axonal transport, reduced axonal dystrophy, decreased Tau neuropathology, and reduced hippocampal neuron loss; however, drug development for AD was discontinued in 2013 after a failed clinical trial [91].

With respect to Tau, additional studies are necessary in order to better understand the exact molecular mechanisms involved in Tau neurotoxicity. Recent studies comparing the neurotoxic profiles of various forms of Tau suggest that a soluble form is likely the most toxic [69]. This has been corroborated by a recent report specifically identifying oligomeric Tau as toxic [92]. Therefore, future therapeutic strategies should be focused on targeting soluble forms of Tau.

### 3.4. Anti-Tau Immunotherapy

Just as with the immunotherapies targeting A*β*, both passive and active immunization approaches against Tau have been considered. In fact, it was demonstrated that reductions in Tau aggregate formation and improved clearance of Tau oligomers and insoluble aggregates could all be achieved with either active or passive immunotherapies [93]. In rodents, treatment with monoclonal antibodies directed against hyperphosphorylated Tau has led to improvements in cognition and was not associated with significant adverse effects [93].

In 2013 Axon Neuroscience began a phase I trial to evaluate the safety and tolerability of AADvac-1, an active immunotherapy which consists of a synthetic peptide derived from the Tau sequence coupled to keyhole limpet hemocyanin; the precise molecular nature of the antigen has not been disclosed (NCT01850238 and NCT02031198) [94]. AADvac-1 uses aluminum hydroxide as an adjuvant. At the 2014 Alzheimer's Association International Conference (AAIC) in Copenhagen, good preclinical safety profile was reported for the treatment period of up to 6 months in rats, rabbits, and dogs. These early results are encouraging and it remains to be seen whether AADvac-1 will demonstrate acceptable safety and efficacy in human patients.

## 4. The Cholinergic Hypothesis

AD is a neurodegenerative disease characterized by a progressive loss of learning and memory as well as neuronal death. The hippocampus, the main brain region involved in memory processing, is influenced by cholinergic modulation [95]. One of the well characterized anomalies associated with neurotransmitter alterations is the degeneration of cholinergic neurons in the nucleus basalis of Meynert and the loss of cholinergic inputs to the neocortex and hippocampus. Several studies reported decreases in choline acetyltransferase (ChAT), acetylcholine (ACh) release, as well as reductions in nicotinic and muscarinic receptors in the cerebral cortex and hippocampus of postmortem AD brains [96]. Acetylcholinesterase inhibitors (AChEI), one of the only 2 classes of drugs currently approved for AD treatment, act by increasing ACh bioavailability at the synapse. Unfortunately, none of these drugs are capable of reversing the course of AD nor of even appreciably slowing down the rate of disease progression [97]. Their clinical effect is largely palliative; however, their potential use in combination therapy with other disease-modifying compounds should not be excluded.

Ladostigil (TV3326) is both a reversible inhibitor of AChE and is a selective and irreversible inhibitor of brain monoamine oxidases A and B, the use of which improves extrapyramidal symptoms and provides an antidepressant effect [98, 99]. It also appears to be a potent antiapoptotic, antioxidant, anti-inflammatory, and neuroprotective agent. NCT01429623 and NCT01354691 phase II clinical trials with ladostigil are currently underway.

## 5. Dendritic Hypothesis (A*β*-PrPC–mGluR5–Fyn Signaling)

Dendritic abnormalities appear in the relatively early stages of AD. While dystrophic neurites, reduced dendritic complexity, and dendritic spine loss are all documented features of AD, it is only recently that we are beginning to understand the underlying molecular changes that occur on the postsynaptic side, in the dendrite [100–102]. Some data suggest that soluble A*β* oligomers are the principal neurotoxic species responsible for dendritic pathology. A*β* oligomers may cause aberrant N-methyl-D-aspartate receptor (NMDAR) activation postsynaptically by forming complexes with the cell-surface prion protein (PrPC). PrPC is enriched at the neuronal postsynaptic density, where it interacts with Fyn tyrosine kinase-metabotropic glutamate receptor 5 complex (Fyn-mGluR5). Fyn activation occurs when A*β* is bound to PrPC-Fyn-mGluR5 complex. Activated in this manner, Fyn can cause tyrosine phosphorylation of the NR2B subunit of this NMDAR. This results in an initial increase and then a loss of cell-surface NMDARs [103]. Fyn overexpression accelerated synapse loss and the onset of cognitive impairment in the J9 (APPswe/Ind) transgenic AD mouse model, while its inhibition produced an opposite effect [100]. In addition, as mentioned earlier, Fyn can also contribute to Tau hyperphosphorylation. Previous studies had reported elevated levels of Fyn in AD brain. Furthermore, Fyn was shown to phosphorylate Tau at Tyr18 residue [101]. Thus, Fyn appears to be a viable target in the treatment of AD pathology. Saracatinib (AZD0530) and masitinib (AB1010) are Fyn kinase inhibitors currently in phase II and phase III clinical trials for mild-to-moderate AD (NCT01864655, NCT02167256, NCT00976118, NCT01872598) [103–105]. Both compounds are capable of blocking Fyn in a nanomolar range.

In a NCT00976118 clinical trial, oral masitinib was administered for a period of 24 weeks, concomitantly with one of the AChEIs (donepezil, rivastigmine, or galantamine) and/or memantine. In that study a significant improvement in the ADAS-Cog test response was reported. These results are encouraging; however, the very small patient pool (*n* = 26) on memantine in this phase II trial is clearly not sufficient to draw conclusions on the potential efficacy of this compound. MOA of masitinib in AD is twofold. Apart from blocking Fyn, masitinib is also a stem cell factor (SCF) receptor (c-KIT) inhibitor. By inhibiting SCF/c-Kit signaling on mast cells (MCs), this compound may prevent neuroinflammation by blocking the activated MCs-microglia interactions [102–105].

## 6. 5-HT6 Receptors in Alzheimer's Disease

5-HT6 receptors are expressed in areas of the CNS involved in learning and memory. Their inhibition was shown to promote acetylcholine release. In AD, 5-HT6 antagonism may lead to the restoration of acetylcholine levels [106]. This hypothesis is supported by evidence that the 5-HT6 receptor antisense oligonucleotides improve spatial learning and memory in the Morris water maze test in normal rats [107]. 5-HT6 inhibitors may be useful in combination therapy, together with AChEIs. For example, Lu-AE-58054 (SGS-518) and PF-05212365 (SAM-531) are being considered as possible treatments for mild-to-moderate AD. Other compounds that are in various stages of clinical research are SUVN-502, AVN-322, and PRX-07034 [108].

## 7. Changing the Concept: AD as a Metabolic Disorder

Clinical studies suggest that diabetes is a major risk factor that contributes to AD pathology. Results from published research indicate that there is a close link between insulin-deficient diabetes and cerebral amyloidosis [109]. Peripheral and central insulin signaling impairments are likely present in both diseases. As a result, “type 3 diabetes” hypothesis of AD was developed, which attempts to bridge the observed metabolic phenotypes present in diabetes and AD into a coherent framework. Insulin hormone is at a centerpiece of this hypothesis [110].

Observations made in the “Hisayama Study” indicate that altered expression of genes related to diabetes mellitus in AD brains may be a result of AD pathology and suggest that peripheral insulin resistance, metabolic syndrome, and/or full-blown diabetes may lead to worsening of cognitive symptoms [111]. Impaired central insulin signaling in the hippocampal circuits, a key region involved in learning and memory, is likely present in AD [112]. Glucose toxicity, insulin resistance, oxidative stress, elevated levels of advanced glycation end products, and cytokine-mediated neuroinflammation are among the proposed mechanisms by which diabetes could increase the risk of AD development. In a recent study, Clarke and colleagues demonstrated that hypothalamic administration of soluble A*β* oligomers initiates neuroinflammatory cascades which eventually cause disturbances in peripheral glucose homeostasis [113]. Tumor necrosis factor *α* (TNF*α*) quite possibly plays an important role in this process [114, 115].

As molecular mechanisms causing AD and T2DM pathologies are possibly related, it is logical to assume that drugs used in T2DM treatment may have a neuroprotective effect in AD [116]. Thiazolidinediones (TZDs) are an example of antidiabetic compounds whose possible role in AD was investigated. TZDs are agonists of peroxisome proliferator-activated receptor *γ* (PPAR-*γ*), which act by promoting PPAR-*γ* heterodimerization with the retinoid X receptor (RXR). PPAR-*γ*/RXR heterodimer is a transcription factor, which regulates expression of genes involved in lipid and glucose metabolism. TZDs improve insulin sensitivity and reduce cytokine-dependent inflammation [117, 118]. Rosiglitazone and pioglitazone are used as antidiabetic drugs, which regulate glucose homeostasis by increasing insulin sensitivity, reducing blood glucose levels, and improving lipid metabolism. Both compounds have also been studied as potential therapeutics for AD treatment, with reported improvements in mitochondrial oxidative metabolism. In animal models, pioglitazone modified various indices of brain aging but did not slow down the cognitive decline [118]. Rosiglitazone and pioglitazone also induce the expression of peroxisome proliferator-activated receptor-*γ* co-activator 1 alpha (PGC-1*α*), a molecule that plays multiple roles in mitochondrial biogenesis, energy metabolism, and mitochondrial antioxidants expression. Previous studies have demonstrated that, in the human brain tissues, the expression of PGC-1*α* decreases with progression of AD dementia [119]. Thus, PGC-1*α* upregulation may improve the mitochondrial energy metabolism and AD pathology [120].

Pioglitazone treatment improved memory and cognition in patients with mild-to-moderate AD in a small clinical trial [121]. A larger phase II trial demonstrated improvements in memory retention and attention with rosiglitazone treatment (6 months) in patients who did not possess an ApoE4 allele [122]. However, phase III trial using rosiglitazone failed to show efficacy in AD (NCT00550420) [123]. It is important to note that in these trials rosiglitazone was administered alone at dosages that were much lower than those needed to exert a beneficial effect on AD pathophysiology in rodent models of the disease. NCT00348140 is a recently completed clinical trial in which rosiglitazone was administrated in combination with AChEIs in patients with mild-to-moderate AD. No results have yet been reported.

Intranasal insulin had also been considered as a treatment option for AD. This particular route of administration is attractive as it bypasses the BBB. This is very significant because insulin transport to the brain from the periphery is dependent on active transport mechanisms which may become disrupted in AD. In addition, the probability of possible adverse events in peripheral tissues is minimized. In theory, insulin delivery directly to the brain will activate cerebral insulin signaling leading to improvements in memory processing and will result in neuroprotection [124, 125]. A currently ongoing clinical trial NCT01767909 is evaluating long-term (12 months) efficacy of intranasal insulin (Humulin R U-100) in mild AD.

Other pancreatic hormones such as amylin may also play a role in AD. Adler and colleagues reported that patients with AD have reduced concentrations of plasma amylin. In transgenic animal models of AD, amylin and pramlintide (amylin analog) decreased brain A*β* levels and improved cognition. Interestingly, amylin also inhibited *β*-secretase enzyme, while pramlintide did not [126, 127].

## 8. Future Strategies

**Figure 1:**
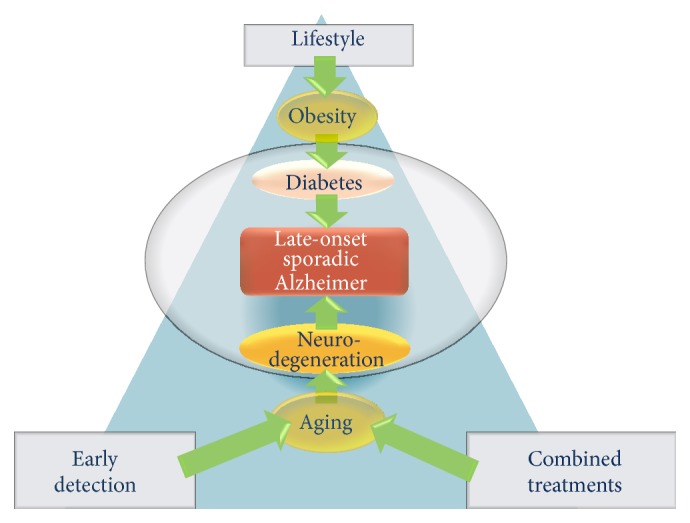
Future strategies in the treatment of late-onset and sporadic forms of AD could be centered on three main points: avoidance of habits and lifestyle leading to obesity and diabetes; early detection of AD biomarkers or structural alterations in presymptomatic individuals; and combined therapies in early phases of cognitive loss.

AD is a complex multifactorial disorder which may require equally complex approaches to treatment. Early disease detection, combination therapies, and lifestyle choices are all likely contributors to the successful eradication of the pathology (Figure 1) [128–132]. A broad range of studies show that inadequate nutrition can increase the risk of disease development [131]. A healthy diet can certainly improve your chances of not developing AD. However, neither the Mediterranean-type diet, caloric restriction, nor antioxidant diet alone can prevent or delay AD. We believe that carefully developed nutrition regimens coupled to combination pharmacotherapies targeting multiple pathways involved in AD are a way forward.

Biomarker identification, indicative of prodromal stages of AD, can lead to early diagnosis and improve prognostic outcomes. Currently existing diagnostic approaches are focused on the detection of A*β*(1-42) and total and phosphorylated Tau levels in the CSF and in the brain. Imaging techniques such as brain MRIs are also used [133–135]. As both A*β* and Tau increases likely appear when the disease had already taken hold, we would welcome the discovery of diagnostic markers which could predict the likelihood of AD development at earlier stages.

Growth factors (GFs) are yet another set of molecules which can potentially improve AD pathology. Transforming growth factor *β* family, insulin-derived GFs (insulin-like growth factor 1, IGF-1 and insulin-like growth factor 2, IGF-2), basic fibroblast growth factor (bFGF), and neurotrophins (nerve growth factor, NGF; brain-derived growth factor, BDGF; glial-derived neurotrophic factor, GDNF) all participate in neurogenesis and neurodevelopment and may be considered as potential targets for AD treatment [135, 136].

## 9. Concluding Remarks

In summary, accumulated evidence suggests that AD neuropathology shows a multifactorial nature and involves multiple biological pathways. Amyloid cascade hypothesis has dominated the field for over 20 years, as a result of which a large number of studies have focused on inhibition and removal of A*β* and senile plaques. Unfortunately, the amyloidocentric approaches have failed to demonstrate improvements in cognition in patients. Dendritic spine defects clearly contribute to cognitive decline observed in AD. These defects are considered an early event in memory circuit's destabilization and should be taken into account for future development of investigational drugs. Novel pharmacotherapies should not be limited to the postulates of the amyloid cascade hypothesis. Events occurring at the synapse may prove to be instrumental in understanding the underlying pathology of this devastating disease.

## Figures and Tables

**Table 1:** 

Activity	Compound	Clinical trial
Inhibitors of *β*-secretase	(i) E2609 (ii) MK-8931 (iii) LY2886721	(i) NCT01600859 (ii) NCT01739348 (iii) NCT01807026 and NCT01561430

Inhibitors and modulators of *γ*-secretase	(i) Semagacestat (LY450139) (ii) Avagacestat	(i) NCT00762411, NCT01035138, and NCT00762411 (ii) NCT00810147, NCT00890890, NCT00810147, NCT01079819

Selective *γ*-secretase modulators (SGSM)	(i) Ibuprofen, sulindac, indomethacin, and R-flurbiprofen (Tarenflurbil) (ii) NIC5-15	NCT00322036, NCT00105547

Nonsteroidal inhibitory of cyclooxygenase activity (NSAIDs)	CHF5074	NCT01203384, NCT01303744, NCT00954252

Inhibitors of A*β* aggregation	(i) Glycosaminoglycans 3-amino acid, 1-propanesulfonic synthetic (3APS, Alzhemed, tramiprosate) (ii) Colostrinin (iii) Scyllo-inositol compound (ELND005) (iv) PBT1 (clioquinol) and PBT2	Phase III in 2007

Modulation of *β*-amyloid transport from the brain to the peripheral circulation	(i) PF-0449470052 (ii) TTP4000 (NCT01548430)	(i) Phase II (ii) Phase I (February 2013)

Active immunotherapy	(i) Anti-A*β*42 vaccine (AN1792) (ii) CAD 106 (iii) ACC-001 (iv) ACI-24, MER5101 and AF205 (v) Bapineuzumab, solanezumab (vi) Gantenerumab (vii) Crenezumab (MABT5102A) (viii) Ponezumab (PF-04360365) (ix) MABT5102A, GSK933776A, NI-101, SAR-228810 and BAN-2401 (x) Gammagard	(i) Phase II (ii) NCT01284387, NCT00479557 and phase II NCT01227564 (rejected) (iii) Preclinical (iv) NCT01127633, NCT02008357 and NCT01900665 phase III (2012) (v) NCT01760005, NCT02051608 and NCT01224106 phase III (vi) NCT01343966, NCT01998841 phase II (April 2013) (vii) Phase I (viii) NCT00818662

**Table 2:** 

Activity	Compound	Clinical trial
Inhibitors of Tau hyperphosphorylation: glycogen synthase kinase 3 inhibitors (GSK3*β*)	(i) Tideglusib (ii) NP031112 (iii) Sodium selenite (VEL015)	(i) NCT01350362 phase II (ii) NCT00948259 (iii) ACTRN12611001200976 phase II
Inhibitors of Tau aggregation	RemberTM, TRx 0237	NCT01626391, NCT01689233, NCT01689246 and NCT01626378
Microtubule stabilizers	(i) Paclitaxel (ii) Epothilone D (iii) TPI 287 (taxane)	(i) Clinical trial 2013 (interrupted) (ii) NCT01966666
Tau-specific immunotherapy	AADvac1 vaccine	NCT01850238 and NCT02031198 phase I trial (2013)

Anticholinesterase inhibitors	(i) Donepezil, rivastigmine, galantamine, (ii) Ladostigil (TV3326)	

PrPC–mGluR5–Fyn signaling	(i) Masitinib (ii) Saracatinib (AZD0530)	(i) NCT00976118 (ii) NCT01864655 and NCT02167256

5-HT6 receptor blockage	Lu-AE-58054 (SGS-518), PF-05212365 (SAM-531), SUVN-502, AVN-322, PRX-07034	Different phases of clinical trials

Antidiabetic drugs	(i) Rosiglitazone and pioglitazone (ii) Intranasal insulin (Humulin R U-100) (iii) Amylin and pramlintide (amylin analog)	(i) NCT00550420, NCT00348140 phase III (ii) NCT01767909 (iii) NCT01429623 and NCT01354691 phase II

Cdk5 inhibitors	Roscovitine and flavopiridol	

## Abbreviations

A*β*: *β*-amyloid
ACE I: Angiotensin converting enzyme I
Ach: Acetylcholine
AD: Alzheimer's disease
ApoE: Apolipoprotein E
APP: Amyloid precursor protein
AB1010: Masitinib
AZD0530: Saracatinib
BACE1: PPAR and aspartyl protease *β*-site A*β*PP-cleaving enzyme
Cdk's: Serine/threonine cyclin dependent
CNS: Central nervous system
CSF: Cerebrospinal fluid
IDE: Insulin degrading enzyme
LRP: Low-density lipoprotein
RemberTM: Methylene blue
MABT5102A: Crenezumab
MAPT: Microtubule-associated protein Tau
MOA: Mechanism of action
MCs: Mast cells
NOS: Nitric oxide synthase
NSAIDs: Nonsteroidal anti-inflammatory drugs
PBT1: Clioquinol
PGC-1*α*: Peroxisome proliferator-activated receptor-*γ* coactivator 1 alpha
PrP: Prion protein
PS1/2: Presenilin 1/2
RAGE: Receptor of advanced glycation end products
ROS: Reactive oxygen species
SGSM: Selective *γ*-secretase modulators of the enzyme
TV3326: Ladostigil
VEL015: Sodium selenite
WHO: World Health Organization.
